# RT-qPCR-Based Estimation of *Phytophthora infestans* Sporangia Using the MFS Transporter Gene PITG_13011

**DOI:** 10.3390/jof12050371

**Published:** 2026-05-17

**Authors:** Hua Zhao, Chunyue Liu, Xi Zhang, Qingfeng Qiu, Yangsheng Luo, Xiwang Ke, Biao Gu

**Affiliations:** 1 State Key Laboratory of Crop Stress Resistance and High-Efficiency Production, Key Laboratory of Plant Protection Resources and Pest Management of Ministry of Education, College of Plant Protection, Northwest A&F University, Yangling 712100, China; zhaohua362@nwafu.edu.cn (H.Z.); chunly0212@163.com (C.L.); 13273462780@163.com (X.Z.); 13262068025@163.com (Q.Q.); 18302902545@163.com (Y.L.); 2 College of Agriculture, Heilongjiang Bayi Agricultural University, Daqing 163319, China; kexylh@163.com

**Keywords:** *Phytophthora infestans*, sporangia-associated marker, RT-qPCR, sporulation, MFS transporter

## Abstract

*Phytophthora infestans* is the causal agent of late blight, one of the most destructive diseases of potato and tomato worldwide. Although qPCR-based methods are widely used to estimate pathogen biomass in infected tissues, methods for specifically assessing sporangial proliferation remain limited. In this study, we developed an RT-qPCR-based assay using *PITG_13011*, which encodes a predicted major facilitator superfamily transporter, as a sporangia-associated molecular marker in *P. infestans*. Among five candidate genes selected from transcriptomic data, *PITG_13011* showed the strongest association with sporangia-associated samples in our validation assays. *PITG_13011* transcripts were detectable from cDNA and genomic DNA derived from as few as 100 sporangia, and transcript abundance showed a strong positive correlation with sporangial number under controlled experimental conditions. In detached leaf inoculation assays, *PITG_13011* transcript levels were associated with differences in sporangia-associated proliferation during infection. These results indicate that *PITG_13011*-based RT-qPCR can serve as a complementary molecular approach for estimating sporangia-associated proliferation of *P. infestans* in laboratory experiments. This method will be useful when sporangial production, rather than total pathogen biomass alone, is the parameter of interest.

## 1. Introduction

Late blight, caused by *Phytophthora infestans* (Mont.) de Bary, remains one of the most destructive diseases affecting potato and tomato production worldwide, causing severe foliar and tuber damage and substantial yield losses [1,2]. As a hemibiotrophic oomycete, *P. infestans* has emerged as a model organism for studying plant-microbe interactions due to its well-characterized genome, experimentally tractable life cycle, and ability to infect both crop hosts and the model plant *Nicotiana benthamiana* [3].

The lifecycle of *P. infestans* consists of six distinct morphological stages [4]: vegetative mycelium (My), zoospore (Zo), cyst (Cy), germinated cyst (Gc), sporangium (Sp), and sporulating mycelium (Spmy). While sexual reproduction produces oospores capable of long-term survival in soil, asexual propagation via sporangia is the primary mode of disease dissemination. Sporangia develop under high-humidity, dark conditions, and their mature forms readily detach from infected tissues to facilitate airborne or waterborne dispersal [5]. This prolific sporangial production is critical for pathogenesis, as each sporangium can release six or more uninucleate zoospores that initiate secondary infection cycles through cyst formation and subsequent penetration of host tissues [4]. Approximately 50% of the *P. infestans* genome exhibits differential expression across lifecycle stages [6], with approximately 30% of genes showing strict stage specificity [7]. These findings highlight the dynamic molecular regulation underpinning morphological changes and host interaction strategies.

Conventional assessment of *P. infestans* infection in laboratory studies relies on three complementary metrics: lesion expansion, biomass accumulation, and sporangial production [8,9,10]. Lesion size remains the most commonly used phenotypic marker for evaluating virulence, as its rate of expansion correlates with host penetration efficiency and colonization success. However, this method has some limitations when comparing the health-disease boundary in early biotrophic phases of oomycetes and minor variations in host susceptibility [8]. To address these challenges, quantifying pathogen biomass via quantitative polymerase chain reaction (qPCR) provides a molecular complement to lesion-based measurements. Biomass determination reveals whether lesion-size discrepancies arise from differential mycelial growth rather than from host-response variability. Combining lesion-area analysis with biomass quantification is often used as an assay for evaluating pathogen virulence and plant resistance in oomycete-plant interactions [3,9,10,11,12]. Light microscopy-based sporangium counting is a method for evaluating a pathogen’s ability to multiply and reinfect by counting the number of sporangia formed in different lesions [9,10]. The speed and number of sporangium formation determine the asexual reproduction, long-distance spread, and reinfection ability of *P. infestans*. Furthermore, knockout of the target gene reduces the number of sporangia, leading to smaller lesions when sporangia are used for inoculation [13,14,15]. A comprehensive consideration of these three indicators can accurately assess changes in virulence and sporulation among diverse isolates and gene-edited transformants. Therefore, developing a molecular assay associated with sporangial proliferation may be useful for controlled studies focused on pathogen reproductive output.

Molecular approaches, such as qPCR assays, offer rapid, specific, sensitive, and high-throughput alternatives to visual-based lesion measurement for pathogen quantification. Many publications have described qPCR assays for oomycete plant pathogens [16,17,18,19]. Molecular markers associated with specific developmental stages may offer a complementary approach for quantifying pathogen proliferation in a more targeted manner. Transcriptome datasets of *P. infestans* have identified genes with elevated expression during sporulation, suggesting the possibility of developing a sporangia-associated molecular assay. However, such candidate genes must be experimentally validated because transcript abundance may vary depending on developmental stage, sample preparation, and experimental conditions.

In this study, we evaluated five candidate genes with elevated transcript abundance during sporulation and identified *PITG_13011* as the most suitable marker in our validation assays. We then established an RT-qPCR-based method to assess *PITG_13011* expression in relation to sporangial number and tested its performance in controlled infection experiments. Compared with microscopy-based sporangia counting, this assay provides a complementary molecular approach for estimating sporangia-associated abundance in experimental samples.

## 2. Materials and Methods

### 2.1. Pathogen and Plant Cultivation

The *P. infestans* strain Pi009 was maintained on RSA (rye sucrose agar) medium in the dark at 16 °C. Stage-specific *P. infestans* propagules were prepared as previously described by Judelson et al. [6] with minor modifications. *P. infestans* samples used for expression analysis included vegetative mycelia, zoospore suspension preparations, and sporangia-associated samples collected from cultures or infected tissues at the indicated developmental stages. Briefly, nonsporulating mycelium was obtained from RS (rye sucrose) broth statically inoculated with 8–10 pieces of 2-day-old mycelium agar plug for 2 days. Sporulating mycelium was obtained by inoculating 25 mL of clarified RS broth into a 90 mm plastic Petri dish with 10^4^ sporangia and culturing for 5 days. Sporangia were isolated by flooding 8- to 12-day-old 150 mm RSA plates with water, rubbing the sporangia free with a glass rod, and then separating sporangia from hyphal debris by passage through 50-μm-pore-sized nylon mesh. Zoospores were produced by incubating sporangia at 4 °C for 60 min, filtered through a 10-μm mesh to remove sporangia, and pelleted by centrifugation at 5000× *g* for 5 min. Cysts were induced by vortexing zoospores for 1 min, then incubating them in RS at 16 °C for 6–8 h. *Solanum tuberosum* desiree and *N. benthamiana* plants (6–8 weeks old) were grown under controlled conditions (14 h light/25 °C, 10 h dark/22 °C) in commercial potting mix. Detached leaves were inoculated by dipping in zoospore suspensions (10^3^ spores/mL), incubated in sealed humidity chambers at 16 °C until sampling at the indicated time points.

### 2.2. Vector Construction and Transient Expression in Plants

Binary vectors encoding green fluorescent protein (GFP), PiAvr3b-GFP, and PiAvr3bM-GFP fusions were constructed using the pMDC backbone as described by Gu et al. [9].

*Agrobacterium tumefaciens* strain GV3101 harboring pMDC-GFP, pMDC-GFP-PiAvr3b, or pMDC-GFP-PiAvr3bM vectors was cultured in LB medium containing 50 μg/mL rifampicin and 50 μg/mL kanamycin at 28 °C with shaking at 200 rpm for 36 h. Cells were pelleted by centrifugation at 5000× *g* for 10 min, resuspended in infiltration buffer (10 mM MES, 10 mM MgCl_2_, pH 5.7) containing 150 μM acetosyringone, and incubated at room temperature for 3 h to induce vir gene expression. Bacterial suspensions were adjusted to OD_600_ = 0.1 using infiltration buffer, and 1 mL syringes were used to infiltrate the abaxial surface of 6-week-old *N. benthamiana* leaves. Infiltrated plants were maintained in a growth chamber (22 °C, 16 h light/8 h dark) for 24 h to allow transgene expression, followed by challenge inoculation with *P. infestans* zoospores (10^3^ spores/mL). Disease symptoms were documented 5–7 days post-inoculation (dpi) using a Canon EOS 70D camera (Ōita, Japan).

### 2.3. Marker Genes Screen and Primer Design

**Figure A1:**
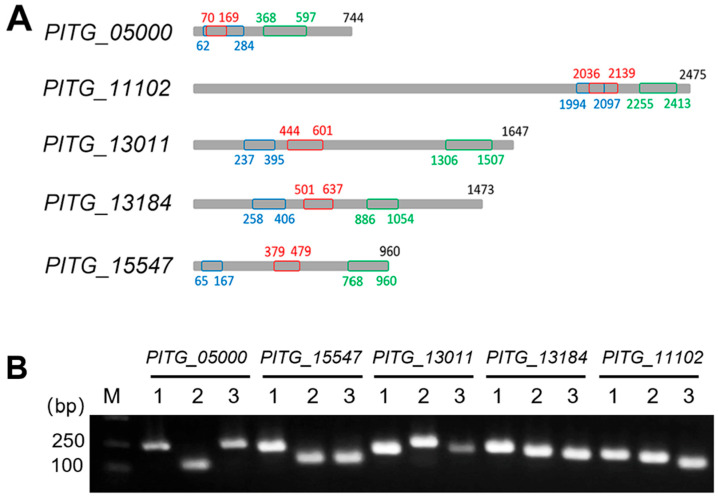
Targeting sites of designed primers of candidate marker genes and the specificity of PCR amplification. (**A**) The length of the coding region of the candidate markers was indicated in base pairs (bp). Blue, red, and green represent three different primer pairs. The number labels the initiation and termination sites of amplification products. (**B**) Amplification specificity detection of selected primers. Run a 1.5% (*w*/*v*) agarose gel at 150 V for 30 min and take pictures with a gel imager. M, molecular weight marker; 1, 2, 3 indicates three pairs of primes targeting different regions of the five marker genes.

Five *P. infestans* genes (*PITG_05000*, *PITG_11102*, *PITG_13184*, *PITG_13011*, and *PITG_15547*; Table A1) were selected based on transcriptomic data indicating stage-specific expression during sporulation. Full-length coding sequences (CDS) were retrieved from the FungiDB database (https://fungidb.org, accessed on 20 May 2024) for subsequent primer design (Figure A1).

**Figure A2:**
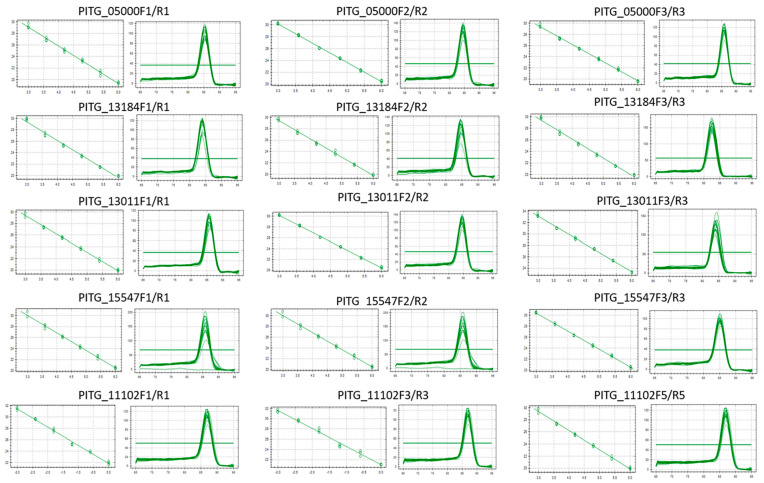
Amplification curve plot (**Left**) and melting curve plot (**Right**) of candidate marker genes.

Three primer pairs were designed per gene using Primer3Plus with the following parameters: 20–25 bp length, 58–62 °C annealing temperature, and 100–200 bp amplicon size. A total of 15 primer pairs were generated (Table A2). Specificity was confirmed via in silico BLAST (v. 2.17.0) analysis against the *P. infestans* T30-4 and *N. benthamiana* reference genomes, and PCR amplification (Figure A1). Oligonucleotide quality was evaluated using the OligoAnalyzer Tool (Integrated DNA Technologies, Coralville, IA, USA) to assess melting temperature, GC content, and secondary structure formation. Validated primers were synthesized by Tsingke Biotechnology Co., Ltd. (Beijing, China). The amplification efficiency and melt curve for qPCR are shown in Figure A2 and Table A2, respectively.

### 2.4. DNA and RNA Isolation and cDNA Synthesis

*P. infestans* samples and infected plant tissues were ground in liquid nitrogen using a mortar and pestle. Genomic DNA (gDNA) was extracted using a DNeasy Plant Pro Kit (Qiagen, Hilden, Germany) with on-column RNase A digestion followed by silica-membrane purification. Final elution was done with 100 μL dH_2_O. DNA concentration was measured using a NanoDrop 2000 spectrophotometer (Wilmington, DE, USA) and adjusted to 400 ng/μL for further applications. To isolate small amounts of gDNA, 100 μL of spore suspension (containing 10 to 1000 sporangia) was added to a 2 mL microcentrifuge tube with two 3 mm stainless-steel beads. The 2 mL tubes, placed in adapter racks, were frozen in liquid nitrogen and homogenized twice (1 min per cycle at 30 Hz) using a Mixer Mill MM400 (Retsch, Haan, Germany). Then, 100 μL of Buffer ATL, 10 μL of proteinase K, and 200 μL of Buffer AL (with 1 μg of carrier RNA) were added, followed by vortexing for 20 s. The lysate was transferred to a fresh 1.5 mL tube, incubated at 56 °C for 15 min, and mixed with 200 μL ethanol. The entire lysate was transferred to a QIAamp MinElute column (QIAGEN N.V., Venlo, The Netherlands), and the procedure was performed according to the manufacturer’s instructions. One-third of the 30 µL elution was used as a PCR template.

Total RNA was extracted from frozen samples using the RNeasy Plant Mini Kit (Qiagen, Hilden, Germany) according to the manufacturer’s protocol. On-column DNase digestion was performed using RNase-Free DNase Set (Qiagen) to remove gDNA contamination. RNA integrity was verified by agarose gel electrophoresis, and concentration was measured with a NanoDrop 2000. The first-strand complementary DNA (cDNA) was synthesized from 1 μg total RNA using the SuperScriptTM III Reverse Transcriptase Kit (Thermo Fisher Scientific, Waltham, MA, USA) with oligo(dT)20 and random primers. cDNA was diluted 1:4 with nuclease-free water before use in qPCR assays.

### 2.5. PCR, Semi-RT-PCR, and RT-qPCR

PCR and semi-RT-PCR were carried out in 20 μL reactions containing 10 ng gDNA and 1 µL of 4-fold diluted cDNA template, 10 µL 2 × M5 Hiper Plus Taq HiFi PCR Mix (Tiangen, Beijing, China), and each gene-specific primer. Thermal cycling was conducted on a Bio-Rad T100^TM^ Thermal Cycler (Hercules, CA, USA) with the following reaction program: 95 °C for 3 min, followed by 35 cycles of a three-step procedure (30 s at 95 °C, 30 s at 56 °C, and 30 s at 72 °C), and 72 °C extension for 5 min. Specifically, 30 cycles were used in optimization experiments with relatively high template abundance, whereas 35 cycles were used for lower-abundance in planta samples to improve detection sensitivity. Quantitative reverse transcription PCR (RT-qPCR) for transcript measurements was performed as described previously using CFX Connect^TM^ Real-Time System (Bio-Rad, Hercules, CA, USA) [20]. The reaction mix contained 1 μL cDNA template, 10 μL SYBR^TM^ Green Universal Mix (Thermo Fisher Scientific), and 0.5 μM primers, for a total volume of 20 μL. Cycling conditions: 95 °C for 1 min, followed by 40 cycles of 95 °C for 15 s and 60 °C annealing and extension for 30 s, 40 cycles; heating from 65 °C to 95 °C at 0.5 °C per 5 s. The standard curve was generated using 4-fold serial dilutions of sporangial gDNA. Ct values were plotted against log-transformed sporangial counts to determine linearity and amplification efficiency. Relative gene expression levels were calculated using the 2^−ΔΔCt^ method with *PiActin02* (*PITG_02389*) as the reference gene. Negative controls (no template) were included in all runs to monitor contamination.

### 2.6. Zoospore or Sporangia Inoculation and Sporangium Counting

Seven-day-old *P. infestans* cultures grown on rye medium in complete darkness were flooded with 2–3 mL pre-cooled sterile water. Plates were then incubated at 4 °C for 2 h to induce zoospore release. After incubation, the sporangial suspensions were filtered through 50-μm nylon mesh to remove mycelial debris. The zoospore concentration was determined using a hemocytometer under light microscopy and adjusted to 100 zoospores/μL with sterile water. For inoculation, 10 μL of zoospore suspension (100 zoospores/μL) was pipetted onto the detached leaf abaxial surfaces from 6-week-old *N. benthamiana* plants. Inoculated detached leaves were maintained in a growth chamber (25 °C, 14/10 h light/dark cycle) for 5–7 days. Lesion development was documented daily using a digital caliper, and final lesion size (mm^2^) was recorded at 7 dpi.

The sporangia suspension was prepared as described by Judelson et al. [6], with minor modifications. Briefly, for the cultures, sporulated hyphae of *P. infestans* grown on 150 mm plates were flooded with 5 mL of sterile distilled water (ddH_2_O). The mixture was then filtered through a 50 μm mesh, centrifuged at 5000× *g* for 5 min, and the resulting pellet was resuspended in sterile ddH_2_O. For the infected leaves: leaf discs were submerged in 5 mL sterile ddH_2_O in a 50 mL Falcon tube. The tube vortexed for two 30 s sets before being filtered, centrifuged, and resuspended. The concentration of sporangia was determined by serial dilution and hemocytometer counting. Inocula of 20 μL containing 10, 100, 1000, and 10,000 sporangia, respectively, were applied to potato leaves, followed by RNA isolation.

### 2.7. Biomass Measurement and Trypan Blue Staining

Relative biomass was estimated using a dual-reference gene normalization approach as described by Gu et al. [9]. Quantitative PCR (qPCR) was conducted with *PiActin02* for *P. infestans* biomass normalization and *NbActin* (*AY594294*) for host plant RNA input normalization.

Leaf samples were stained in trypan blue solution (10 mL lactic acid, 10 mL glycerol, 10 g phenol, 10 mg trypan blue dissolved in 10 mL ddH_2_O) and incubated at 65 °C for 2 h in a water bath. After staining, samples were destained sequentially: Chloral hydrate was added, and the leaves were left at room temperature for 2–6 h. The chloral hydrate was replaced once and left overnight. The chloral hydrate was removed, and 70% glycerol was added. The leaves were observed and photographed under a microscope. For correlation analysis, samples used for manual sporangia counting and RT-qPCR were collected from the same biological material or from parallel samples prepared under identical experimental conditions.

## 3. Results

### 3.1. Identification of Sporangia Marker Genes and Expression Profile

To identify molecular markers associated with sporangial development in *P. infestans*, we selected five candidate genes (*PITG_05000*, *PITG_11102*, *PITG_13011*, *PITG_13184*, and *PITG_15547*) from published transcriptomic datasets [7], based on their elevated transcript abundance during sporulation (Table 1).

**Figure 1:**
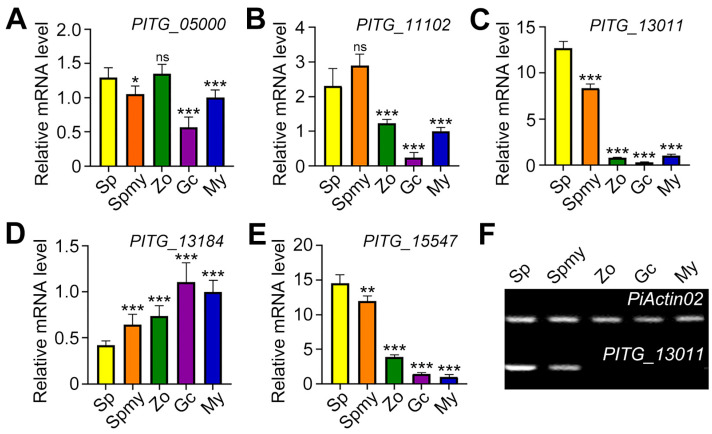
Expression pattern of candidate marker genes in various asexual development stages. (**A**–**E**), Reverse-transcribed quantitative polymerase chain reaction (RT-qPCR)-based analysis of the expression levels of *PITG_05000*, *PITG_11102*, *PITG_13011*, *PITG_13184*, and *PITG_15547* in *P. infestans* samples from artificial media. Complementary DNA (cDNA) was synthesized from the total RNA of *P. infestans* samples. Sp: Sporangia; Spmy: Sporulating mycelium; Zo: Zoospore; Gc: Germinated cyst; My: Mycelium. Error bars represent the standard deviation of three technical replicates. This experiment was replicated three times. (**F**) Expression pattern of marker gene *PITG_13011* using semi-quantitative PCR with the same samples as in (**A**–**E**). The amplification cycle is 30, and the PCR products are run on a 1.5% agarose gel to check the results. *PiActin02* was used as a reference gene (*** *p* < 0.001, ** *p* < 0.01, * *p* < 0.05, ns not significant).

RT-qPCR analysis across six lifecycle stages (sporangium, sporulating mycelium, zoospore, germinated cyst, and mycelium) revealed differential expression patterns. As shown in Figure 1A,D, *PITG_05000* and *PITG_13184* failed to show significant upregulation in sporangia compared to mycelium stages. *PITG_11102* and *PITG_15547* showed increased transcript levels in zoospores (Figure 1B,E), compromising their specificity for sporangial quantification. *PITG_13011* transcript abundance was markedly higher in sporangia-associated samples than in the other tested stages (Figure 1C), supporting its use as a marker for sporangial proliferation under our experimental conditions. Semi-RT-PCR confirmed this pattern, detecting *PITG_13011* transcripts exclusively in sporangial and sporulating mycelial tissues (Figure 1F). Although not all candidates showed expression patterns fully consistent with previously published transcriptomic data, *PITG_13011* displayed the clearest and most reproducible enrichment in sporangia-associated samples in our experiments. We therefore selected *PITG_13011* for further analysis.

### 3.2. Detection Limit Analysis of PITG_13011 Transcripts

**Figure 2:**
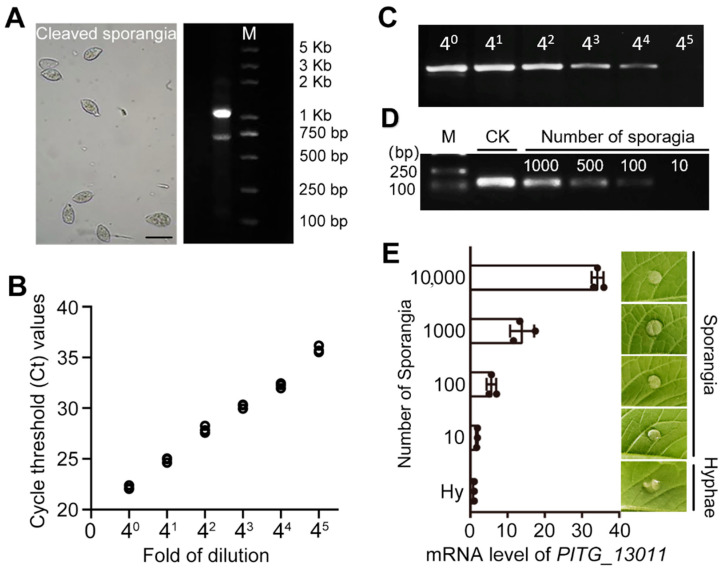
Sensitivity of *PITG_13011* transcripts in detecting *P. infestans* sporangia. (**A**) Microscopic observation of representative *P. infestans* sporangia undergoing cleavage after maturation (**left**), quality assessment of total RNA from sporangia using agarose gel electrophoresis (**right**), scale bar 50 μm. (**B**,**C**), Amplification sensitivity of *PITG_13011* transcripts by serial dilution (4-, 16-, 64-, 256-, and 1024-fold) of cDNA template synthesized from 1 μg of total RNA, detected by qPCR and semi-qPCR. Experiments were performed in triplicate. (**D**) Amplification sensitivity of *PITG_13011* from genomic DNA (gDNA) of different amounts of sporangia. PCR was performed for 40 cycles, and products were analyzed on a 1.5% agarose gel. Lanes 1–5 correspond to molecular weight markers (M), and samples containing 1000, 500, 100, and 10 sporangia, respectively. This experiment was replicated twice. (**E**) Expression levels of *PITG_13011* in different numbers of sporangia inoculated onto the abaxial surface of Desiree potato leaves, and total RNA was extracted immediately after inoculation.

To eliminate variation due to RNA extraction from a small number of sporangia, we performed a serial dilution of cDNA to determine the detection limits of *PITG_13011* transcripts. A total of 200 μg of RNA was isolated from 5.8 × 10^9^ mature sporangia that were cleaved from sporulating mycelium, with quality verified by agarose gel electrophoresis (Figure 2A). Reverse transcription of 1 μg RNA generated cDNA that was serially diluted 4–4^5^ fold (4×, 16×, 64×, 256×, 1024×). Semi-quantitative PCR and qPCR analysis revealed that *PITG_13011* amplicons remained detectable at a 256-fold dilution, but were undetectable at a 1024-fold dilution. The qPCR Ct values exceeded 35 at 1024-fold dilution, indicating unreliable amplification (Figure 2B,C). *PITG_13011* transcripts could be effectively detected from 256× diluted cDNA, suggesting that this RT-qPCR method is sufficient to detect mRNA variations derived from sporangia on leaf lesions, which generally form 10^4^–10^5^ sporangia [9,10]. In addition, we confirmed the detection limit of *PITG_13011* in gDNA using templates from suspensions containing 1000, 500, 100, and 10 sporangia. As shown in Figure 2D, PCR products were obtained from the templates with 100 sporangia, while no amplification was observed from templates with only 10 sporangia.

To replicate the natural infection process in which sporangia land on potato leaf surfaces and initiate infection, potato leaves were inoculated with varying numbers of sporangia. RNA was extracted from the inoculated samples before zoospore release. Following reverse transcription into cDNA, quantitative PCR (qPCR) was performed to detect the *PITG_13011* gene, which serves as a reliable indicator of sporangial quantity on leaves. As shown in Figure 2E, *PITG_13011* was consistently detected in samples containing 100 sporangia, while its detection was substantially reduced in samples with only 10 sporangia. These results indicate that *PITG_13011* can be detected from few-sporangia samples and is suitable for molecular estimation of sporangia-associated abundance in controlled experiments.

### 3.3. PITG_13011 Transcript Abundance Correlates with Sporangial Number

**Figure 3:**
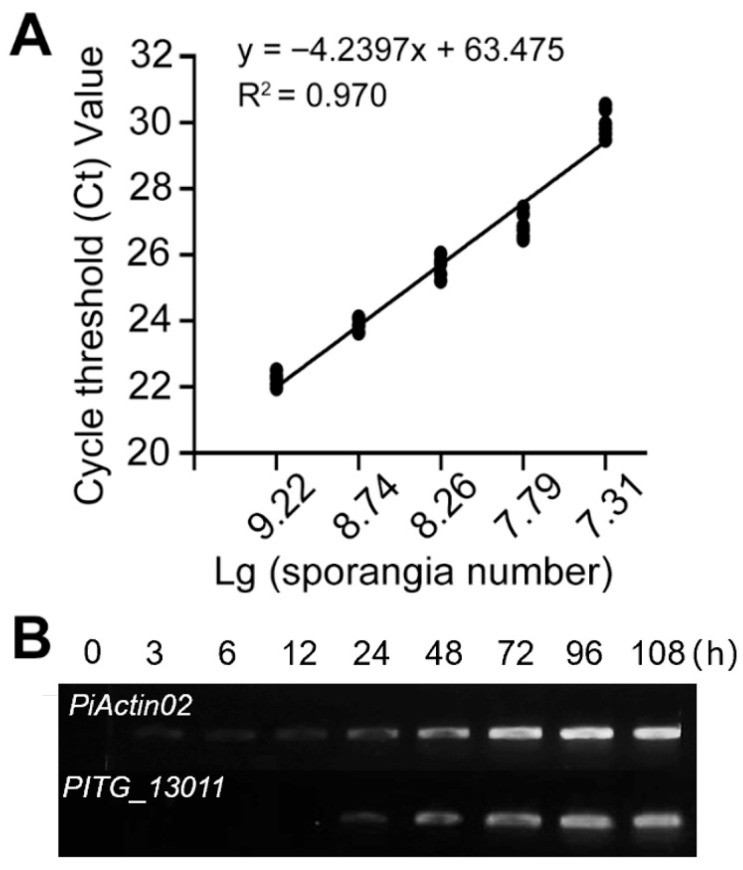
Time-course analysis of *PiActin02* and *PITG_13011* transcript abundance during *P. infestans* infection of *N. benthamiana* leaves. (**A**) The horizontal axis is the base ten logarithm of the sporangia number, and the vertical axis is the qPCR cycle threshold (Ct) value. Statistical analysis is based on the F test; the adjusted R^2^ is 0.960, *p* = 0.0022. (**B**) The expression profile of *PiActin02* and *PITG_13011* during *P. infestans* infection. *PiActin02* was used as a pathogen-associated reference, whereas *PITG_13011* was evaluated as a sporangia-associated marker over a 108 h time course on *N. benthamiana* leaves using semi-RT-PCR. The amplification cycle is 35, and the PCR products are run on a 1.5% agarose gel for analysis.

To validate the linearity of the qPCR assay, 1.92 × 10^9^ sporangia were serially diluted 2×, 4×, 8×, and 16×. cDNA prepared from these samples was diluted 4× and analyzed via qPCR to establish the relationship between the log10-transformed sporangial number and *PITG_13011* expression. The experiment yielded regression coefficients of R^2^ = 0.970 (adjusted R^2^ = 0.960), confirming a high degree of linear correlation (*p* < 0.0022, F test) (Figure 3A). These results demonstrate that sporangial densities, as determined by RT-qPCR, correlate with manual counts under a light microscope.

To further monitor the new sporangia formation in planta, *N. benthamiana* leaves were inoculated with zoospore suspensions (10^3^ spores) to avoid pre-existing sporangia interference on *PITG_13011* transcripts abundance. RNA was prepared at 0 h, 3 h, 6 h, 12 h, 24 h, 48 h, 72 h, 96 h, and 108 h post-inoculation (hpi), reverse-transcribed into cDNA, and analyzed by qPCR. *PITG_13011* transcript levels increased as infection progressed and were associated with subsequent sporangial proliferation in lesions (Figure 3B). This temporal pattern correlated with the sporangial-formation dynamics observed via trypan blue staining, in which mature sporangia were abundant on leaf surfaces from 72 to 108 hpi under laboratory conditions.

### 3.4. Analysis of the Number of Sporangia During Lesion Development

**Figure 4:**
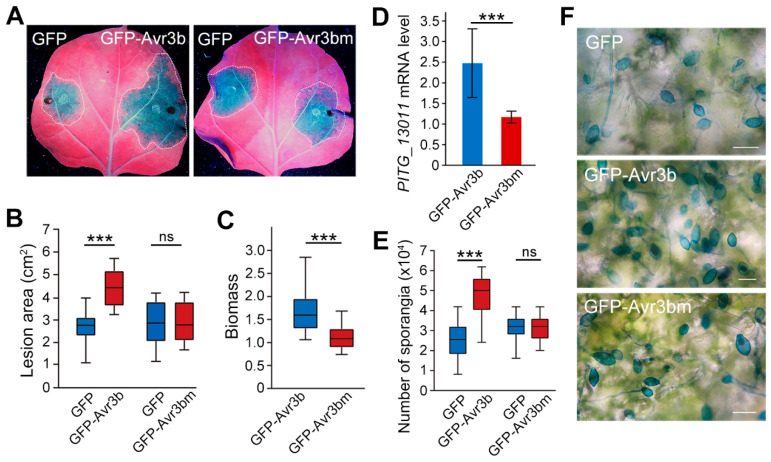
Quantitative analysis of plant susceptibility after transient expression of target genes. (**A**) Disease symptoms of *P. infestans* zoospores infecting *N. benthamiana* leaves that transiently expressed control GFP (left) and GFP fusion of *P. infestans* RxLR effector PiAvr3b or non-functional mutant PiAvr3bm (right) (OD_600_ = 0.2). Pictures were taken 5 days post-inoculation. At least 10 leaves were tested in each independent experiment. The experiment was repeated three times with similar results. Dotted lines indicate lesion edges. (**B**) Quantification of lesion sizes shown in (**A**). Pooled data from three independent experiments that comprise 10 inoculations are presented as mean ± SD. (**C**) Quantitative measurement of relative pathogen biomass shown in (**A**). *P. infestans* biomass in *N. benthamiana* leaves expressing GFP, GFP-PiAvr3b, or GFP-PiAvr3bm was determined via quantitative PCR and normalized to the *NbActin* genes. (**D**) Numbers of sporangia isolated from diseased tissues shown in (**A**). (**E**) The relative expression level of *PITG_13011* at 5 days after *P. infestans* zoospore inoculation; (**F**) Trypan blue stain of sporangia formed on diseased tissues, scale bar 50 μm. Paired Student *t* test, *** *p* < 0.001, ns not significant.

To validate the utility of *PITG_13011* for quantifying sporangial proliferation in planta, we analyzed the correlation between the expression level of *PITG_13011* and the sporangia formation during lesion development. We employed *P. infestans* RxLR effector PiAvr3b and its mutant PiAvr3bm to generate the variation in host susceptibility by transiently expressing them in *N. benthamiana* leaves. Forty-eight hours after agroinfiltration, the detached *N. benthamiana* leaves were inoculated with *P. infestans* zoospores (10^3^ per droplet). At five dpi, PiAvr3b-expressing leaves exhibited significantly larger lesions compared to GFP controls (Figure 4A,B). Pathogen biomass (quantified by *PiActin02* qPCR) showed a 1.59-fold increase in PiAvr3b compared to GFP (Figure 4C). RT-qPCR analysis demonstrated 2.47-fold upregulation of *PITG_13011* in PiAvr3b-expressed tissues relative to GFP controls (Figure 4D). Sporangia counts from lesion-containing leaf discs revealed a 2-fold increase in PiAvr3b tissues versus GFP. In contrast, the non-functional mutant PiAvr3bm showed no significant difference from GFP in lesion size, sporangia count, or *PITG_13011* expression (Figure 4E,F). Taken together, our results indicated that *PITG_13011*-based RT-qPCR can provide a complementary molecular readout in infection assays when reproductive output is of interest.

## 4. Discussion

In this study, we established an RT-qPCR-based assay using *PITG_13011* as a sporangia-associated molecular marker for *P. infestans.* In our validation experiments, *PITG_13011* showed a stronger association with sporangia-associated samples than the other candidate genes tested, and its transcript abundance was positively correlated with sporangial number. These findings support the use of *PITG_13011* as a molecular indicator of sporangia-associated proliferation under controlled laboratory conditions. A key feature of this assay is that it addresses a parameter distinct from total pathogen biomass. Many published qPCR methods for oomycete pathogens are designed to estimate overall pathogen colonization in host tissues. By contrast, the present assay is intended to provide a readout associated with sporangial production, which is more directly related to asexual reproductive output. This method can be complementary to lesion measurement and biomass quantification.

At the same time, the present study has several limitations. First, the assay was validated only in *P. infestans*, under laboratory conditions. Second, although *PITG_13011* showed a strong association with sporangia-associated samples in our experiments, we did not exhaustively test cross-reactivity against other *Phytophthora* species or host genomic DNA. Third, our validation scope was limited to the experimental systems examined here, and broader testing across additional isolates, host genotypes, and environmental conditions will be required before wider application can be considered.

Our results also underscore the importance of experimentally validating candidate marker genes derived from transcriptomic data. Although the published dataset provided a useful starting point [7], not all selected genes showed identical expression behavior in our RT-qPCR assays. Such differences may reflect variation in developmental staging, experimental conditions, analytical methods or *P. infestans* isolates. Because zoospores served as the inoculum and may also be released from newly formed sporangia during lesion expansion, *PITG_15547* was not considered an appropriate marker for assessing changes in sporangial abundance due to its elevated expression in zoospores. Consequently, *PITG_13011* was selected based on its reproducible performance in validation experiments. Importantly, *PITG_13011* expression increased dramatically as sporangial numbers rose during infection, further supporting its suitability as a marker for monitoring changes in sporangial abundance.

The biological basis of *PITG_13011* upregulation during sporulation remains unclear. *PITG_13011* encodes a predicted major facilitator superfamily transporter, but its specific role in sporangial development has not yet been determined. Future work examining the functional contribution of *PITG_13011* to sporulation or sporangial physiology may provide further insight into why this gene performs well as a sporangia-associated marker. The MFS is a widely distributed and conserved family in fungi and oomycetes [21]. It was reported that *Peronophythora litchi* MFS1 transcripts were enriched in both sporangia and oospores, as well as during the infection process [22].

The amplification sensitivity of *PITG_13011* primers was evaluated by performing PCR amplification using gDNA and cDNA derived from sporangia. PCR successfully detected both gDNA and cDNA samples equivalent to as few as 100 sporangia. Furthermore, cDNA reverse-transcribed from sporangial RNA remained detectable by qPCR after a 256-fold dilution. Collectively, these results show that the assay performed reliably over the sporangial range tested, rather than indicating that at least 10,000 sporangia are required for detection. Under experimental conditions, lesions on *N. benthamiana* leaves generally contained approximately 10,000 to more than 100,000 sporangia at 3 to 7 dpi [9,10]. Accordingly, this assay may provide a complementary molecular readout for analyzing relatively large differences in sporangia-associated proliferation under controlled conditions. In addition, transcript signals were detectable in infected potato leaf tissue containing approximately 100 sporangia, indicating a sensitivity comparable to that reported by Llorente et al. [18], whose qPCR assay used DNA of 250 sporangia as the amplification template.

The quantitative analysis of *PITG_13011* expression levels revealed a strong positive correlation between *PITG_13011* transcript abundance and sporangial number, with an adjusted regression coefficient (R^2^) of 0.960, during asexual development and infection. This underscores the potential to analyze diseased samples and distinguish sporulation variations among gene-edited transformants and chemically treated *Phytophthora* pathogens under controlled conditions. Nonetheless, factors such as variable DNA extraction efficiency and potential inhibition by plant compounds may affect the accuracy of quantification.

*PITG_13011*-based RT-qPCR provides a useful molecular approach for estimating sporangia-associated proliferation of *P. infestans* in controlled experiments. This assay may be valuable when the biological question concerns pathogen reproductive output in addition to lesion development or total pathogen biomass. Future validation across broader experimental settings will determine the full scope and limitations of this method. Due to the conservation of the *PITG_13011* gene in *Phytophthora* pathogens (Table A3), this method may be applied and extended to other *Phytophthora*-host interaction systems once the specific expression pattern of homolog genes is validated. This is particularly relevant for *Phytophthora* species, such as *P. sojae* and *P. capsici*, whose sporangia do not fall off, necessitating measurement of their numbers by staining and microscopic observation. Taken together, the present work provides proof of concept for the feasibility of this approach under controlled experimental conditions; however, further validation is required before it can be considered for routine diagnostic use.

## Figures and Tables

**Table 1:** Annotation information of candidate marker genes.

Gene ID	Name	Homologous Genes	Length
*PITG_05000*	MtN3-like protein	24	744 bp
*PITG_11102*	cGMP-dependent protein kinase	5	2745 bp
*PITG_13011*	Sporangia induce major facilitator superfamily	4	1647 bp
*PITG_13184*	Mucolipin-like protein	2	1473 bp
*PITG_15547*	Amino acid polyamine organocation Family	4	960 bp

## Data Availability

The original contributions presented in this study are included in the article. Further inquiries can be directed to the corresponding author.

## Appendix A

**Table A1:** Primer sequences.

Name	Forward Primer	Reverse Primer
*PITG_05000*F1/R1	CGACTGCTCAACTCCAAGAA	CATGACCACACCCAGAGAAT
*PITG_05000*F2/R2	CCAGATTTCCGACGCATTCA	CATAGGAGCCCACAACACG
*PITG_05000*F3/R3	CGCCGTATCCAGATTTCCGA	TTTAGTGTGGTCCGATGGGC
*PITG_15547*F1/R1	TCGAGTGATGTTGGTGGCTC	CTACCGGATTGTACCCGTCG
*PITG_15547*F2/R2	ACCAAGTGGATCAGCCGAAG	AGTGCCGCAGTCACGAAATA
*PITG_15547*F3/R3	GAGAACGAGACGCCCTATGG	AACAGATTTTCACCCACCGC
*PITG_13011*F1/R1	GCTGGTTCTCCATTCCTCCT	GTACCGTTCGAGAGCCGTAG
*PITG_13011*F2/R2	AGGTATTGTTGGTGCGGGAG	CGTGGGTGCTAATCCTCCAT
*PITG_13011*F3/R3	TCGTGACGAGTTACACTGGC	AGCACCCAGATTTCCCCATC
*PITG_13184*F1/R1	GCGAGTACCATGTGGAGCTT	GTGTTCCCCAGTTGAGCGTA
*PITG_13184*F2/R2	GACGGCGGTTCACTACCTAC	GCGTTTCTAAATCGCCCTGTG
*PITG_13184*F3/R3	CGCCCTCAAGTTTTCGTTCC	CTTCGCAGTCCTCCAGTTGT
*PITG_11102*F1/R1	AACCCAGCACTGCGTATCG	CATCGAAGTTGCCAGCATCG
*PITG_11102*F3/R3	TATGCAGGACGTGGTGAACC	TCGAATCTTCGTGTCCTCCG
*PITG_11102*F5/R5	CCGAGCTTGTGTTAAACAAAGG	ATTGTGCTGGAATGGAGTGC

**Table A2:** Amplification efficiency of primers of candidate marker genes.

Primers	Amplicon Length	Amplification Efficiency
*PITG_05000*F1/R1	230 bp	102.8%
*PITG_05000*F2/R2	100 bp	103.8%
*PITG_05000*F3/R3	223 bp	103.9%
*PITG_11102*F1/R1	166 bp	105.6%
*PITG_11102*F3/R3	157 bp	92.4%
*PITG_11102*F5/R5	104 bp	109.2%
*PITG_13184*F1/R1	169 bp	102.5%
*PITG_13184*F2/R2	149 bp	104.4%
*PITG_13184*F3/R3	137 bp	104.7%
*PITG_13011*F1/R1	159 bp	107.0%
*PITG_13011*F2/R2	202 bp	104.2%
*PITG_13011*F3/R3	158 bp	102.3%
*PITG_15547*F1/R1	193 bp	106.6%
*PITG_15547*F2/R2	103 bp	103.0%
*PITG_15547*F3/R3	101 bp	101.7%

**Table A3:** Homologous genes of *PITG_13011* in oomycetes.

Strains	Homologous Genes	Gene ID	Evalue
*Phytophthora infestans T30-4*	4	*PITG_13011*	0
		*PITG_13008*	0
		*PITG_09342*	1 × 10^−115^
		*PITG_13010*	2 × 10^−118^
*Phytophthora cactorum*	3	*PC110_g12657*	0
		*PC110_g12653*	0
		*PC110_g12656*	0
*Phytophthora capsici*	6	*DVH05_027226*	0
		*DVH05_027223*	0
		*DVH05_027221*	0
		*DVH05_027218*	0
		*DVH05_027222*	0
		*DVH05_027220*	0
*Phytophthora cinnamomi*	5	*IUM83_02887*	0
		*IUM83_02880*	0
		*IUM83_02890*	0
		*IUM83_02888*	0
		*IUM83_02889*	0
*Phytophthora palmivora var. palmivora strain sbr112.9*	2	*PHPALM_799*	0
		*PHPALM_6669*	0
*Phytophthora parasitica INRA-310*	4	*PPTG_16902-t1*	0
		*PPTG_16902_t2*	0
		*PPTG_16903*	0
		*PPTG_16904*	0
*Phytophthora ramorum 14567*	10	*KRP22_10808*	0
		*KRP22_11592*	0
		*KRP22_11594*	0
		*KRP22_10810*	0
		*KRP22_10812*	0
		*KRP22_11596*	0
		*KRP22_10809*	0
		*KRP22_10811*	0
		*KRP22_11593*	0
		*KRP22_11595*	0
*Phytophthora sojae strain P6497*	4	*PHYSODRAFT_520413*	0
		*PHYSODRAFT_317695*	0
		*PHYSODRAFT_520735*	0
		*PHYSODRAFT_317694*	0
